# Prevalence and molecular characterization of human bocavirus in children with acute gastroenteritis in Beijing, China, during 2014–2023

**DOI:** 10.1128/spectrum.03327-24

**Published:** 2025-04-25

**Authors:** Zeng Li, Kexiang Zhang, Ri De, Zhenzhi Han, Yanpeng Xu, Liying Liu, Liping Jia, Huijin Dong, Hui Huang, Li Deng, Linqing Zhao

**Affiliations:** 1 Laboratory of Virology, Beijing Key Laboratory of Etiology of Viral Diseases in Children, Capital Institute of Pediatrics 36776, Beijing, China; 2 Department of Infectious Disease, Children's Hospital Affiliated to Capital Institute of Pediatrics, Beijing, China; Changchun Veterinary Research Institute, Chinese Academy of Agricultural Sciences, Changchun, China

**Keywords:** children, acute gastroenteritis, human bocavirus, prevalence, evolution

## Abstract

Human bocavirus (HBoVs) is an emerging virus globally, and its prevalence, diversity, and evolution in children with acute gastroenteritis require further study. Fecal specimens collected from outpatients with acute gastroenteritis in Beijing, China, from April 2014 to December 2023, were tested for HBoVs by PCR targeting the NS1 gene. Genotyping was based on the NP1/VP1 boundary region, and nearly full-length HBoV2 sequences were analyzed phylogenetically. A total of 79 HBoVs-positive specimens (2.5%, 79/3,116, 95% CI: 2.0%–3.1%) were detected, with HBoV2 (65.8%, 52/79, 95% CI: 55.4%–76.3%) as the dominant genotype, followed by HBoV1 (25.3%, 20/79, 95% CI: 15.7%–34.9%) and HBoV3 (8.9%, 7/79, 95% CI: 2.6%–15.1%). HBoVs were distributed throughout the year, with higher positive rates observed from August to December 2018–2021. They predominantly infected children younger than 5 years, especially those aged 6–24 months (3.3%, 52/1,591, 95% CI: 2.4%–4.1%). HBoV2C was the prevalent sub-genotype in Beijing. Novel recombination events were detected between HBoV2 sub-genotypes, with breakpoints in the NS1 and VP3 gene regions. Evolutionary analysis estimated the time to the most recent common ancestor (tMRCA) for HBoV2 dating back to 1845, with a mean nucleotide substitution rate of 1.4 × 10^−4^ substitutions/site/year, and that HBoV2A evolved faster than HBoV2C. The evolutionary rates decreased in the following order: NP1, VP3, VP1, and NS1. A purifying selection was observed on HBoV2 genes, with one positively selected site in the NS1 gene. In conclusion, HBoV2 was the dominant genotype in children with acute gastroenteritis in Beijing, China, with higher susceptibility in those under 2 years old. Novel recombination events between HBoV2 sub-genotypes occurred frequently.

**IMPORTANCE:**

Acute gastroenteritis remains a leading cause of morbidity and mortality in children, with viral infections being the primary causative agents. In this study, we investigated the prevalence of human bocavirus (HBoVs) in children with acute gastroenteritis in Beijing from 2014 to 2023, identifying HBoV2C as the predominant sub-genotype. Additionally, this study reported the first estimate of the evolutionary rate for global HBoV2 (1.4 × 10^−4^ substitutions/site/year) and identified novel intra-genotype recombination events in HBoV2. The results not only filled a gap in the evolutionary studies of global HBoV2 but also offered valuable data for the development of effective surveillance and prevention strategies for controlling acute gastroenteritis in children.

## INTRODUCTION

Acute gastroenteritis is a leading cause of morbidity and mortality in children worldwide, ranking as the third most common cause of death in children under 5 years of age. Annually, there are approximately 1.7 billion cases of childhood acute gastroenteritis, resulting in 443,832 deaths in children under five (1). Viruses are the leading causative agents of acute gastroenteritis, including rotavirus, calicivirus, enteric adenovirus, and astrovirus, affecting patients of all ages, particularly children (2). With the development of advanced gene sequencing methods, several emerging viruses have been identified in the feces of pediatric cases of acute gastroenteritis. However, the epidemiological characteristics and disease mechanisms of these viruses are still to be clarified through further studies (3).

Human bocavirus 1-4 (HBoV1-4), as emerging viruses, have been detected in both the respiratory and fecal specimens, and HBoV1 has been identified as the causative agent for acute respiratory infections in children (4). However, the clinical implications of HBoVs in acute gastroenteritis have long been questioned despite their common detection in fecal specimens from pediatric cases of acute gastroenteritis, which has garnered widespread attention (5).

HBoV is a member of the *Parvoviridae* family, within the *Bocaparvovirus* genus, with a small, non-enveloped, icosahedral virus with a linear, single-stranded DNA molecule of approximately 5.5 kb in length. Its genome comprises three major open reading frames that encode non-structural proteins, the nuclear phosphoprotein (NP1), and viral capsid proteins (VP1–VP3) (6). HBoVs are considered highly diverse and frequently recombinant viruses. On the basis of the principle proposed by Kapoor et al., HBoV strains exhibiting >8% protein and >10% nucleotide divergence in the VP1 gene should be classified as distinct species, while those showing >1.5% protein and >5% nucleotide divergence should be classified as different genotypes. Based on VP1 gene divergence, HBoVs have been classified into four genotypes (HBoV1-4) (7), and HBoV2 was subdivided into clusters A, B, and C (8). Intergenotype recombination has been observed in all four human bocaviruses, and a recombination event between HBoV1 and a common ancestor of HBoV2 and HBoV4 may have resulted in the formation of HBoV3 (9). Based on the analyses of the HBoV2 genome, intra-genotype recombination between HBoV2A and HBoV2B led to the formation of HBoV2C (10). It has been reported that the recombination breakpoint occurred at the NP1 and VP1 gene boundary region, and thus, the region was frequently employed for genotyping (7, 10).

So far, all four genotypes of HBoVs have been detected in fecal specimens of children with acute gastroenteritis (5). The epidemiology of HBoVs causing gastroenteritis has been studied worldwide. A systematic review and meta-analysis of 36 studies found that the pooled prevalence of HBoVs among 20,591 patients with acute gastroenteritis was 6.9% (11). Meanwhile, in China, the positive rate of HBoVs in fecal specimens of children with acute gastroenteritis ranged from 2.2% to 20.4%, and HBoV2 exhibited higher prevalence and genetic diversity in fecal specimens than the others (12–14). Additionally, it has been reported that a higher load of HBoV2 may indicate an increased risk of acute gastroenteritis in children (15). Children younger than 5 years are more susceptible to HBoVs (16). HBoVs have been detected throughout the year in fecal specimens, with seasonality according to geographic location, and they tend to peak in the summer and autumn in China (14).

However, limited data are available on the prevalence and genetic characteristics of HBoVs in children with acute gastroenteritis over a long period in Beijing, China. This study aimed to describe the prevalence, evolution, and clinical manifestations of HBoVs in pediatric patients with acute gastroenteritis in Beijing, China, from 2014 to 2023.

## MATERIALS AND METHODS

### Clinical specimens

Between April 2014 and December 2023, fecal specimens were gathered from outpatients suffering from acute gastroenteritis at the Affiliated Children’s Hospital of Capital Institute of Pediatrics. The inclusion criteria in this study were (i) children under 15 years, (ii) excreting feces three or more times a day for less than 2 weeks, and (iii) with liquid, watery, or mucous feces. The exclusion criteria were (i) patients diagnosed with chronic gastroenteritis for more than 2 weeks and (ii) with insufficient feces available for HBoV detection (17). The fecal specimens were diluted with phosphate buffer at a 1:10 ratio, mixed using a vortex, and then centrifuged at 1,500 × *g* for 15 minutes. Supernatants were gathered into sterile tubes and kept at −20°C.

### Nucleic acid extraction

The QIAamp MinElute Viral Spin Kit (Qiagen, Germany) was used to extract nucleic acid from fecal supernatants, following the instructions provided by the manufacturer.

### Screening for HBoVs and genotype identification by PCR

To screen for HBoVs, the primers HBoV2-sf2 and HBoV2-sr2 were employed to amplify a 495-nucleotide segment within the NS1 gene of HBoVs in clinical specimens, as previously described (10).

To genotype, a 690-nt fragment at the NP1/VP1 boundary region was amplified from HBoV-positive specimens using primers HBoV-c1 (5′-CTTYGAAGAYCTCAGACC-3′) and HBoV-c2 (5′-TKGAKCCAATAATKCCAC-3′) designed from GenBank sequences of HBoV1-4^12^. Then, PCR products were analyzed by electrophoresis on a 1.5% (wt/vol) agarose gel. The amplicons with the expected size were then sent to SinoGenoMax (Beijing, China) for sequencing using the ABI 3730xl DNA Analyzer (Thermo Fisher Scientific, USA). Chromas software was used to evaluate the quality of the raw sequencing data, while assembly and editing of sequences were performed using EditSeq and SeqMan software from DNASTAR. The BLAST tool (https://blast.ncbi.nlm.nih.gov/Blast.cgi) was used to compare all nucleotide sequences with reference strains from the NCBI GenBank database. For those sequences with high quality, phylogenetic analysis was performed to get the phylogenetic tree.

### Near full-length genome of HBoV2 amplified and sequenced

For specimens positive for HBoV2, nearly full-length HBoV2 genomes were amplified from HBoV2-positive specimens and sequenced via Sanger sequencing using primers developed by Zhao et al. (Table S1) (18). A comprehensive phylogenetic analysis and investigation of recombination events in HBoV2 were subsequently conducted.

### Phylogenetic analysis

The phylogenetic trees were built using sequences from this study along with HBoV1-4 reference sequences from GenBank (Table S2). Multiple-sequence alignments were conducted by MAFFT v7.520. Sequence identities were compared using BioAider v1.727 (19). The suitable nucleotide substitution model was selected by ModelFinder (20). Using the maximum-likelihood method with 1,000 bootstrap replications, phylogenetic trees were built with IQ-TREE v2.2.2.6 (21) and visualized through the online tool tvBOT (22).

### Evolutionary analysis

A Bayesian Markov Chain Monte Carlo (MCMC) method, implemented in Beast v1.10.4, was applied to infer the time-resolved phylogenetic tree of nearly full-length HBoV2 genome sequences along with the evolutionary rates of the coding regions (23). The best-fitting model was selected using ModelFinder, and the optimal combination of clock models and tree priors was determined by comparing the Bayes Factor across different model combinations (Table S3). MCMC chains were run for a minimum of 100,000,000 iterations, with sampling occurring every 10,000 generations. Convergence was evaluated by checking the effective sample size using Tracer v1.7.2 after discarding the first 10% as burn-in, with only values exceeding 200 being considered. The uncertainty in the estimated evolutionary rates was quantified using 95% highest posterior density (HPD) intervals. The maximum clade credibility (MCC) tree was generated using TreeAnnotator v1.10.4, with common ancestor heights derived following the 10% burn-in phase. Finally, trees were visualized using FigTree v1.4.4.

### Recombination analysis

To identify recombination events, the Recombination Detection Program 5 (RDP5) was utilized, incorporating seven distinct methods: RDP, GENECONV, Chimaera, MaxChi, Bootscan, SiScan, and 3Seq, with a threshold *P*-value of 0.05 (24). Recombination events were considered valid only if at least three of the methods detected them, ensuring the robustness of the results. The suspected recombinant sequences were further validated using Simplot software, which performs similarity and bootscanning analyses with a sliding window of 200 bp and a step size of 20 bp. Recombinant validation was performed by analyzing the flanking gene sequences at the recombinant site through phylogenetic tree construction. This thorough approach provides a reliable framework for evaluating recombination events within the genetic data.

### Selective pressure analysis

Selective pressure on sites was evaluated using the ratio (ω) of non-synonymous substitutions (dN) to synonymous substitutions (dS), employing several algorithms, including single likelihood ancestor counting (SLAC), fixed effects likelihood (FEL), mixed effects model of evolution (MEME), and fast unconstrained Bayesian approximation (FUBAR) through the Datamonkey online platform (http://www.datamonkey.org/) (25). Neutral evolution was defined as ω = 1, negative selection as ω < 1, and positive selection as ω > 1. The study assessed potential positively selected (PSS) and negatively selected sites (NSS) in the NS1, NP1, and VP1 genes of HBoV2. Sites were classified as positively selected if at least two algorithms showed *P*-values less than 0.05 for SLAC, FEL, and MEME, and a Bayes factor or posterior probability greater than 0.95 for FUBAR.

### Statistical analysis

The basic clinical data of pediatric patients positive for HBoVs, including gender, age, and diagnosis, were collected. All statistical analyses were conducted using SPSS software, version 25.0. The distribution of variables was assessed with the Shapiro-Wilk test. The 95% confidence intervals (95% CIs) for the detection rates were calculated using the normal approximation method or the Clopper-Pearson method. Patient profiles by age were described using the median and interquartile range (IQR). The Chi-square test was applied to evaluate the statistical significance of HBoVs-positive rates across genders and age groups. A *P*-value < 0.05 was considered statistically significant.

## RESULTS

### Prevalence of HBoVs

In this study, 3,116 specimens were collected from children with acute gastroenteritis in Beijing, China, between 2014 and 2023. The male-to-female ratio was 1.60:1 (1,919:1,197), and the median age was 0.83 years (IQR: 0.43–1.67).

There were 79 fecal specimens (2.5%, 79/3,116, 95% CI: 2.0%–3.1%) positive for HBoVs in the study determined by PCR to amplify NS1 gene with the ratio of male to female 46 (2.4%, 46/1,919, 95% CI: 1.7%–3.1%):33 (2.8%, 33/1,197, 95% CI: 1.8%–3.7%) (*χ^2^* = 0.39, *P* = 0.53). More were shown in 2014 (3.8%, 19/506, 95% CI: 2.1%–5.4%), then in 2015 (3.6%, 18/500, 95% CI: 2.0%–5.2%), 2017 (3.2%, 9/281, 95% CI: 1.1%–5.3%), 2016 (2.5%, 12/475, 95% CI: 1.1%–3.9%), 2019 (2.1%, 6/285, 95% CI: 0.4%–3.8%), 2018 (2.1%, 2/97, 95% CI: 0.3%–7.3%), 2021 (2.0%, 8/407, 95% CI: 0.6%–3.3%), 2023 (1.1%, 3/281, 95% CI: 0.2%–3.1%), 2020 (1.0%, 1/105, 95% CI: 0%–5.2%), and 2022 (0.6%, 1/179, 95% CI: 0%–3.1%) (Table 1).

**TABLE 1:** Yearly distribution of specimens positive for HBoVs and different genotypes in children with acute gastroenteritis from 2014 to 2023 in Beijing, China

Year	Number of specimens tested	Number of specimens positive for HBoVs (%)	Number of specimens and proportion (%) of different genotypes		
			HBoV1	HBoV2	HBoV3
2014	506	19 (3.8)	4 (21.1)	11 (57.9)	4 (21.1)
2015	500	18 (3.6)	6 (33.3)	9 (50.0)	3 (16.7)
2016	475	12 (2.5)	2 (16.7)	10 (83.3)	0
2017	281	9 (3.2)	3 (33.3)	6 (66.7)	0
2018	97	2 (2.1)	1 (50.0)	1 (50.0)	0
2019	285	6 (2.1)	2 (33.3)	4 (66.7)	0
2020	105	1 (1.0)	1 (100.0)	0	0
2021	407	8 (2.0)	1 (12.5)	7 (87.5)	0
2022	179	1 (0.6)	0	1 (100.0)	0
2023	281	3 (1.1)	0	3 (100.0)	0
Total	3,116	79 (2.5)	20 (25.3)	52 (65.8)	7 (8.9)

**Fig 1:**
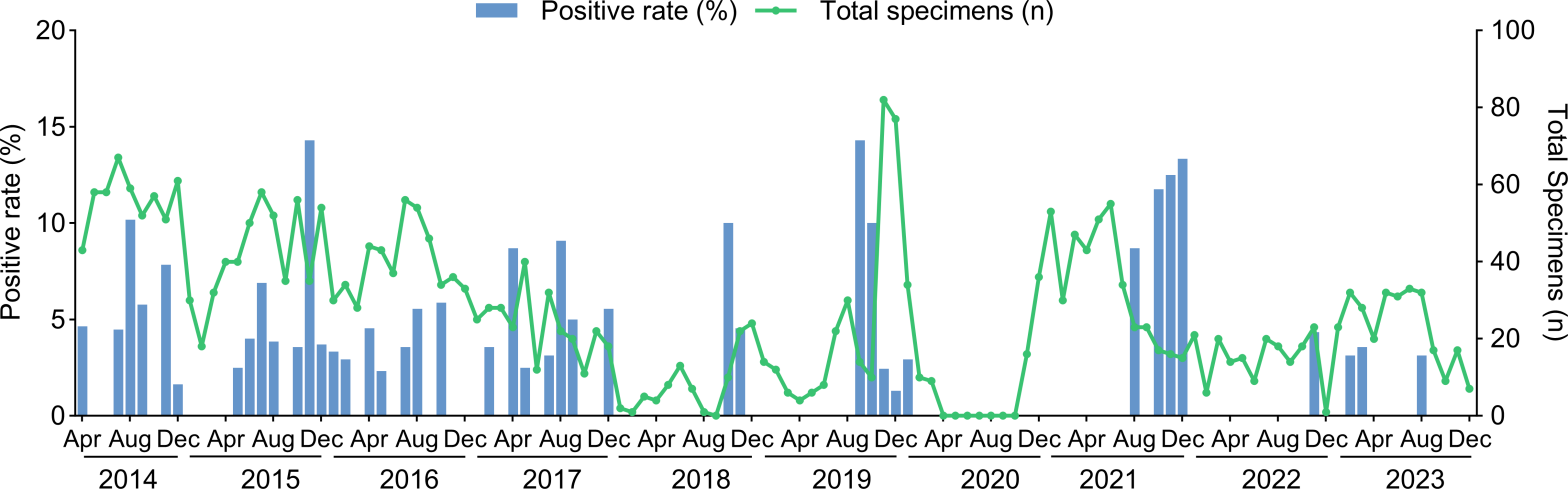
Monthly distribution of specimens positive for HBoVs in children with acute gastroenteritis in Beijing, China, from 2014 to 2023.

The monthly distribution of HBoVs-positive specimens from 2014 to 2023 revealed that there was no obvious seasonal epidemic of HBoVs during 2014–2017, while higher positive rates were shown from August to December in 2018–2021 (Fig. 1).

In four groups of aged <6 months, 6–24 months, 24–60 months, and ≥60 months, the highest positive rate of HBoVs was shown in the group aged 6–24 months (3.2%, 52/1,591, 95% CI: 2.4%–4.1%), followed by that aged 24–60 months (2.3%, 11/482, 95% CI: 0.9%–3.6%), aged <6 months (1.8%, 15/851, 95% CI: 0.9%–2.6%), and ≥60 months (0.5%, 1/192, 95% CI: 0%–2.9%). Significant differences were shown between the group aged 6–24 months and the group aged <6 months (*χ^2^* = 4.71, *P* = 0.03), and the group aged ≥60 months (*χ^2^* = 4.48, *P* = 0.03), respectively.

### Genotyping of HBoVs

The sequences of the NP1/VP1 boundary region from these 79 specimens positive for HBoVs over the past 10 years were clustered into three groups, including 20 (30.2%, 20/79, 95% CI: 15.7%–34.9%) into HBoV1, 52 (65.8%, 52/79, 95% CI: 55.4%–76.3%) into HBoV2 and 7 (8.9%, 7/79, 95% CI: 2.6%–15.1%) into HBoV3, respectively. Sequences of HBoV2 were dominant throughout the whole study period with a positive proportion from 50.0% to 100.0%, except that in 2020 (0 cases), and that of HBoV1 were the second one in consistently positive proportion from 12.5% to 33.3%, except that in 2018 (1 case, 50.0%), 2020 (1 case, 100.0%), 2022 (0 cases), and 2023 (0 cases). However, sequences of HBoV3 were detected only in 2014 and 2015 (Table 1).

In the monthly distribution of specimens positive for different HBoV genotypes, HBoV2 consistently prevailed throughout the year, especially in August (4.1%, 12/291, 95% CI: 1.8%–6.4%), October (3.6%, 8/222, 95% CI: 1.2%–6.1%), and November (2.8%, 9/320, 95% CI: 1.0%–4.6%). HBoV1 was more frequent in July (2.1%, 7/329, 95% CI: 0.6%–3.7%) and November (1.3%, 4/320, 95% CI: 0.3%–3.2%), while the positive rate of HBoV3 was at a low level throughout the year and at higher levels in August (0.7%, 2/291, 95% CI: 0.1%–2.5%) and November (0.6%, 2/320, 95% CI: 0.1%–2.2%) (Fig S1).

In four age groups, HBoV2 was detected in all four age groups with proportions ranging from 61.5% to 100.0%. In contrast, HBoV1 was not detected in children ≥60 months, and HBoV3 was only detected in children younger than 24 months (Fig S2).

**Fig 2:**
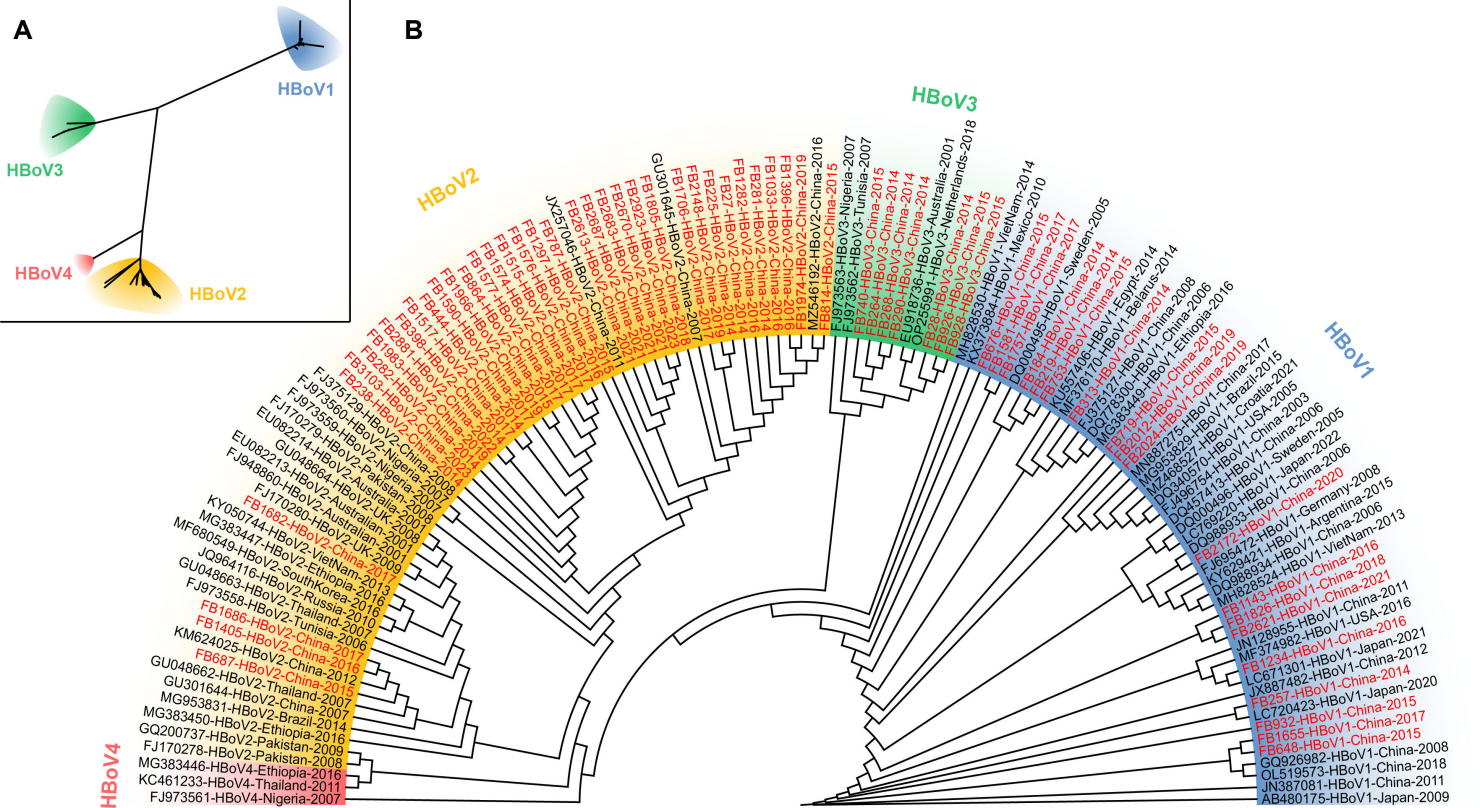
The phylogenetic trees were constructed using 63 sequences of the NP1/VP1 boundary region with high quality and the 62 reference sequences of HBoV1-4 from GenBank, including unrooted tree (**A**) and circular tree (**B**). The analysis was inferred using the maximum-likelihood method. The sequences were labeled with the GenBank accession or laboratory number, genotype, country, and year of collection at each branch, with different background colors: blue for sequences of HBoV1, yellow for those of HBoV2, green for those of HBoV3, and pink for those of HBoV4. The sequences from clinical specimens collected in the study were highlighted in red.

For phylogenetic analysis, 63 sequences with high quality and complete NP1/VP1 boundary region sequences were included, and 16 with partial sequences were excluded (Fig. 2). Among those 63 sequences, 19 were clustered into HBoV1, 37 into HBoV2, and 7 into HBoV3.

### Phylogenetic analysis of the nearly full-length genomes of HBoV2

Among those 37 specimens positive for HBoV2 and with high-quality NP1/VP1 boundary region sequences, there were 15 successfully amplified to generate nearly full-length genome sequences of HBoV2 and submitted to GenBank with accession nos. PQ728256–PQ728270, 12 with only partial genome sequences of HBoV2, and 10 with deficient volumes of specimens. Fortunately, at least one nearly full-length genome sequence of HBoV2 in each year was included in this study (Fig S3).

**Fig 3:**
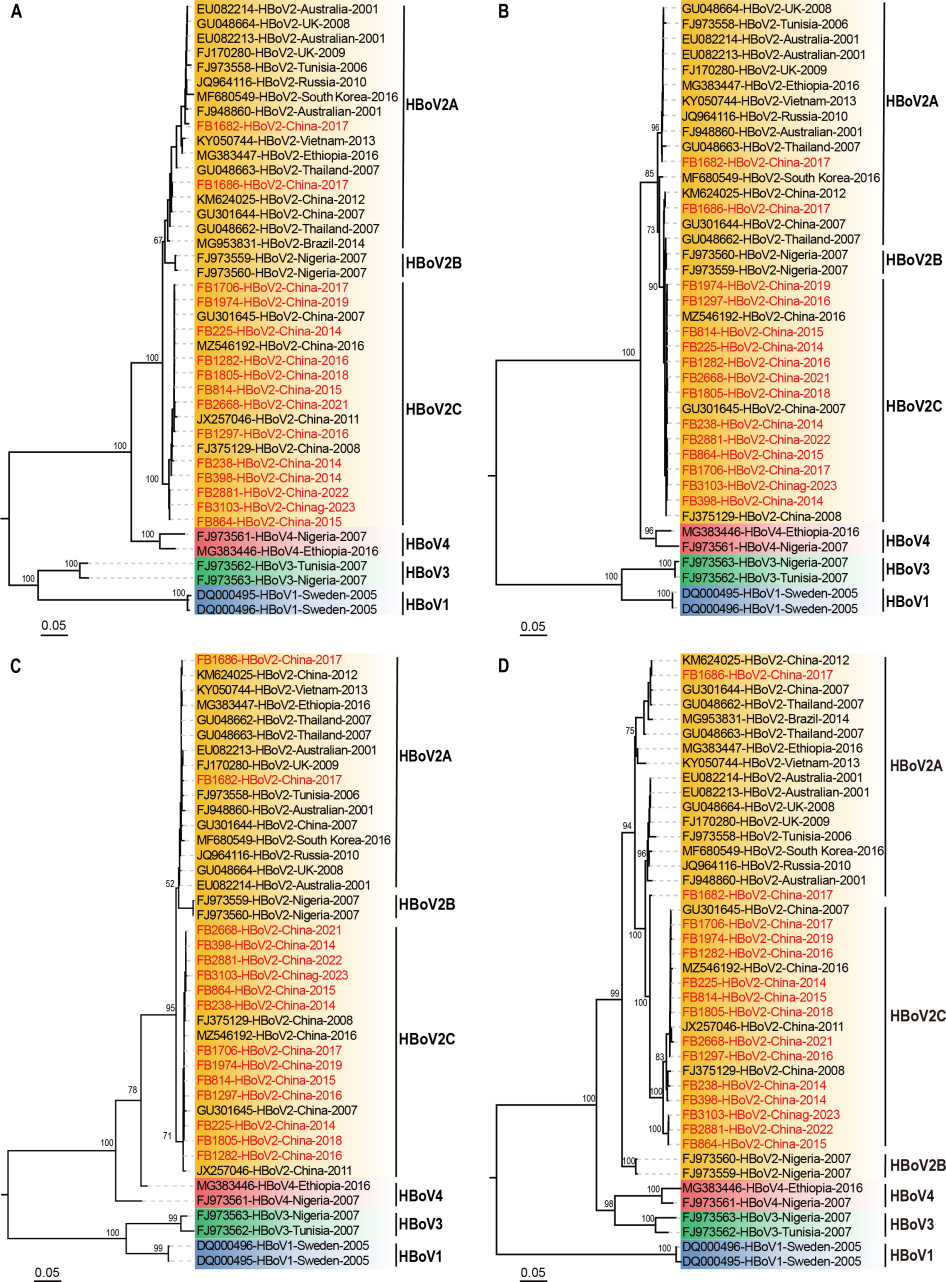
The 15 nearly full-length HBoV2 genome sequences obtained from clinical specimens and the reference sequences from GenBank of HBoV1-4 were used for tree construction. The analysis was inferred using the maximum-likelihood method. Bootstrap support values are indicated at key nodes. The scale bar at the bottom represents the genetic distance. The GenBank accession number or laboratory number, genotype, country, and year of the collection were labeled for each sequence with different genotypes in different background colors, including blue for sequences of HBoV1, yellow for those of HBoV2, green for those of HBoV3, and pink for those of HBoV4. The sequences from clinical specimens collected in the study were highlighted in red. (A) The nearly full-length genome sequences. (B) The NS1 gene sequences. (C) The NP1 gene sequences. (D) The VP1 gene sequences.

These 15 nearly full-length genome sequences of HBoV2 were clustered into two distinct groups, including two (FB1682-HBoV2-China-2017 and FB1686-HBoV2-China-2017) in the cluster of HBoV2A with 96.9%–99.9% identities to reference sequences, and 13 in the cluster of HBoV2C with 98.5%–99.8% identities to reference sequences from China (Fig. 3A). The phylogenetic trees of the coding region of NS1, NP1, and VP1 showed that the cluster of HBoV2C was closer to HBoV2B in NS1 and NP1 gene trees (Fig. 3B and C), and closer to HBoV2A in VP1 gene tree (Fig. 3D). The FB1682-HBoV2-China-2017 was in the cluster of HBoV2A in NS1, NP1 gene trees (Fig. 3B and C), and in the cluster of HBoV2C in VP1 gene tree (Fig. 3D). The FB1686-HBoV2-China-2017 was in the cluster of HBoV2B in the NS1 gene tree, and in HBoV2A in NP1 and VP1 gene trees. These results suggested recombination events to some extent.

**Fig 4:**
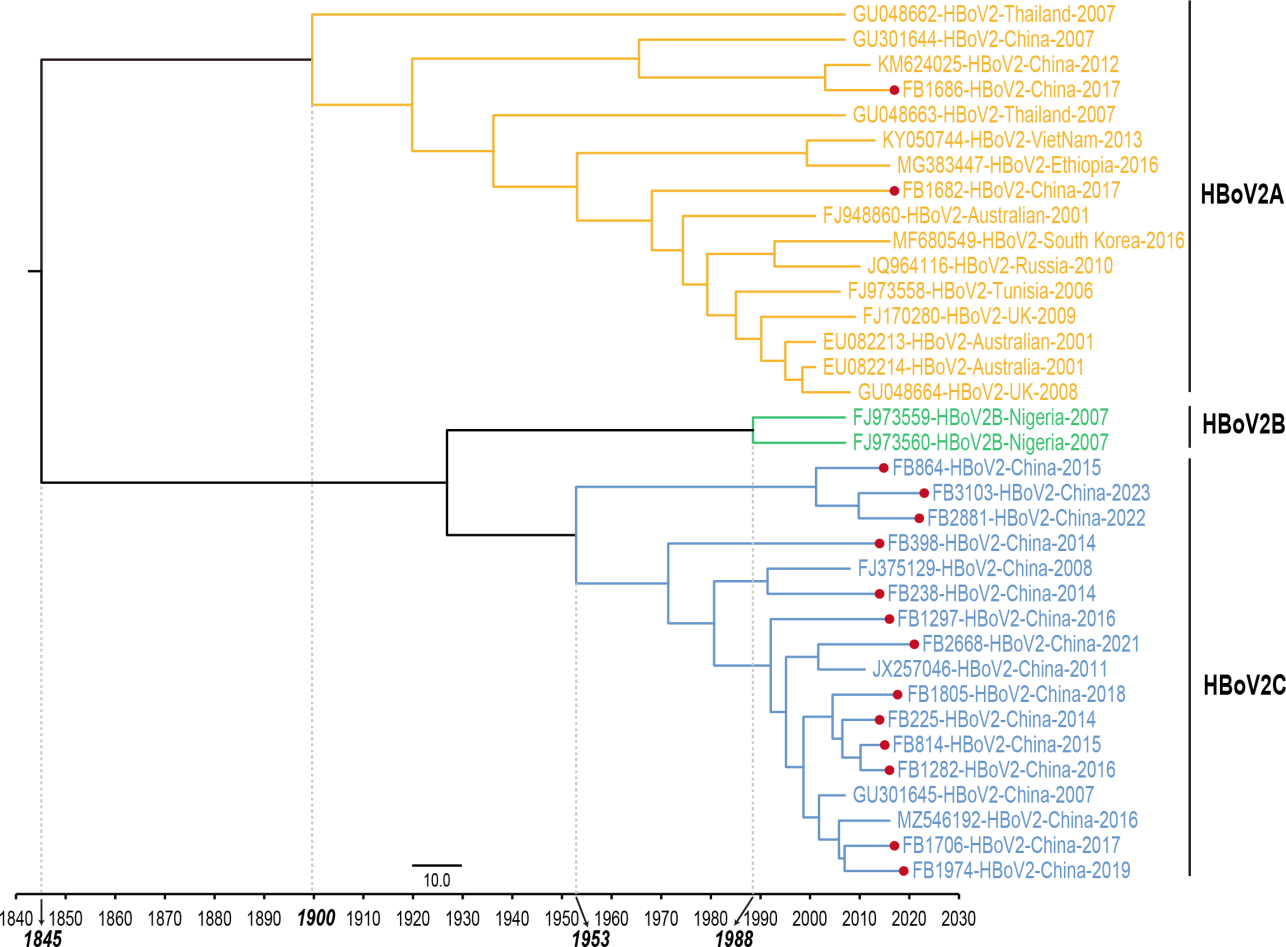
The MCC phylogenetic tree was constructed based on the 15 nearly full-length HBoV2 genome sequences. The sequences were labeled with the GenBank accession or the laboratory number, genotype, country, and year of collection with different clade colors for different clusters, including yellow for HBoV2A, green for HBoV2B, and blue for HBoV2C. The sequences from clinical specimens collected in the study were indicated by red solid circles. The x-axis was the time scale (years).

The MCC tree was constructed using the 15 nearly full-length and the reference HBoV2 sequences with the MCMC method (Fig. 4). The evolutionary substitution rate of the nearly full-length HBoV2 sequences in this study was estimated to be 1.4 × 10^−4^ substitutions/site/year (95% HPD range: 3.5 × 10^−5^–2.7 × 10^−4^). The time to the most recent common ancestor (tMRCA) was calculated to be 1845, after which the lineage diverged into HBoV2A around 1900, with a mean evolutionary rate of 2.0 × 10^−4^ substitutions/site/year (95% HPD: 3.8 × 10^−7^–4.4 × 10^−4^). For HBoV2B and HBoV2C, the tMRCAs were traced back to 1988 and 1953, respectively. The evolutionary rate for HBoV2C, which included 13 nearly full-length HBoV2 sequences in this study, was 1.4 × 10^−4^ substitutions/site/year (95% HPD: 8.6 × 10^−9^–3.0 × 10^−4^), suggesting that HBoV2C evolved at a lower rate than HBoV2A.

The evolutionary rates of NS1, NP1, VP1, and VP3 genes of HBoV2 were 1.3 × 10^−4^ (95% HPD: 5.2 × 10^−5^–1.9 × 10^−4^), 2.4 × 10^−4^ (95% HPD: 2.5 × 10^−6^–4.8 × 10^−4^), 1.5 × 10^−4^ (95% HPD: 3.9 × 10^−5^–2.6 × 10^−4^), and 1.8 × 10^−4^ (95% HPD: 3.1 × 10^−5^–3.6 × 10^−4^) substitutions/site/year, respectively, with the highest evolutionary rates in NP1 gene, and the lowest one in NS1 gene.

### Recombination analysis of HBoV2

Using RDP5 and Simplot software, all sequences of HBoV2C in this study were recombinants of HBoV2B and HBoV2A, as reported in the previous study (10).

**Fig 5:**
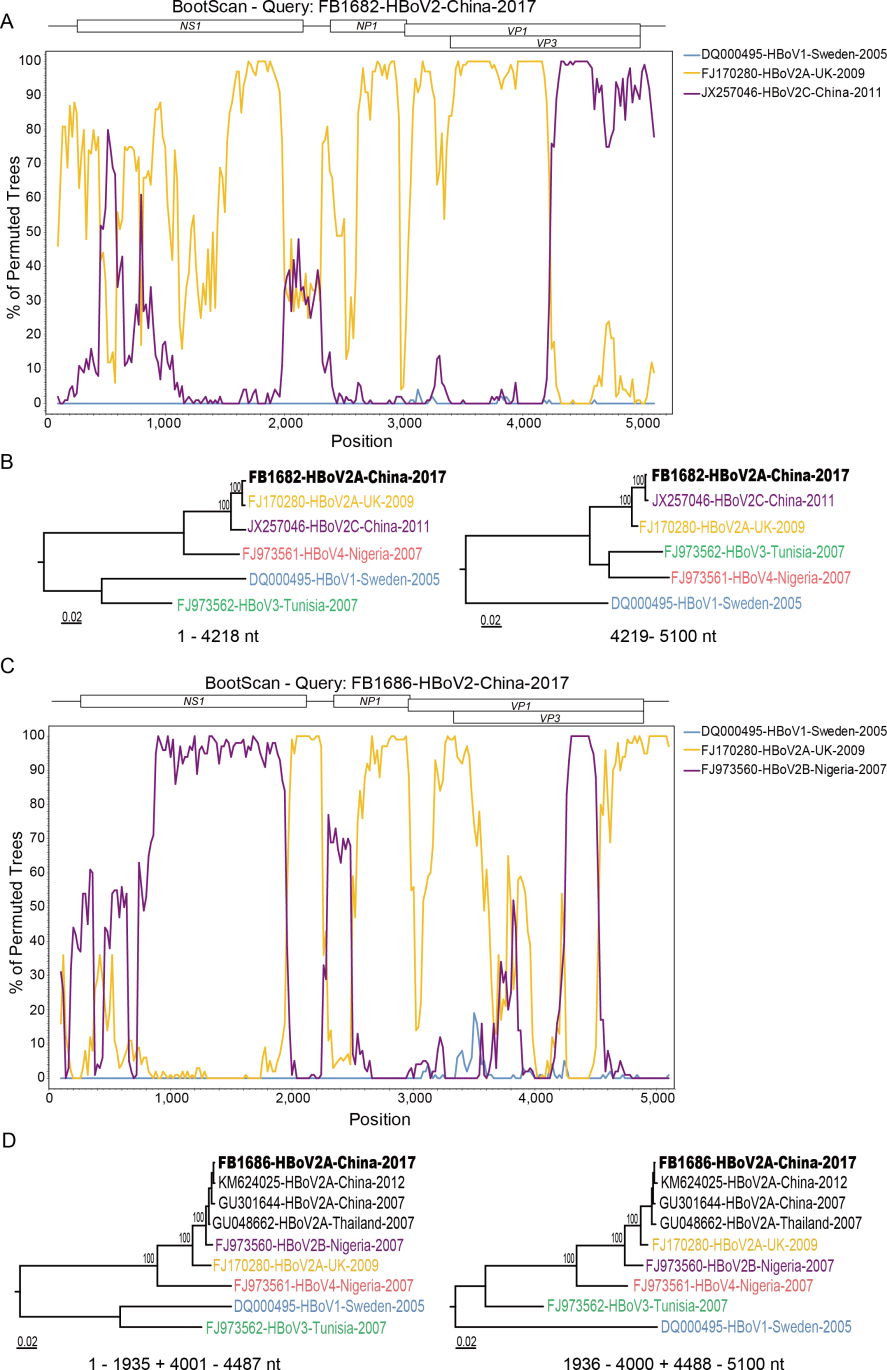
Recombination analyses for the nearly full-length genome sequences of FB1682-HBoV2-China-2017 and FB1686-HBoV2-China-2017 using Simplot software. (A) Bootscanning analysis of the nearly full-length gene sequence of FB1682-HBoV2-China-2017 compared with reference sequences. (B) Construction of phylogenetic trees based on recombination regions and non-recombination regions of FB1682-HBoV2-China-2017 and reference sequences using IQ-TREE. (C) Bootscanning analysis of the nearly full-length gene sequence of FB1686-HBoV2-China-2017 compared with reference sequences. (D) Construction of phylogenetic trees based on recombination regions and non-recombination regions of FB1686-HBoV2-China-2017 and reference sequences using IQ-TREE.

For FB1682-HBoV2-China-2017, the potential major parent was identified as FJ170280-HBoV2A, sharing 99.0% nucleotide identity, while the potential minor parent was identified as JX257046-HBoV2C, sharing 97.5% nucleotide identity. The recombination breakpoint was located on the 4,218 nt, corresponding to the VP3 gene region (Fig. 5A and B). This finding was further confirmed by bootscanning analysis via SimPlot software and the construction of a phylogenetic tree.

For FB1686-HBoV2-China-2017, the potential major parent was identified as FJ170280-HBoV2A, sharing 97.4% nucleotide identity, and the potential minor parent was identified as FJ973560-HBoV2B, sharing 96.7% nucleotide identity. The recombination breakpoints were located on the 1,935 nt, 4,000 nt, and 4,487 nt, corresponding to the NS1 and VP3 gene regions. These results were further confirmed by bootscanning analyses via Simplot software and the construction of a phylogenetic tree (Fig. 5C and D).

Taking into account the intra-genotype recombination events in HBoV2, the evolutionary rates were re-estimated by accounting for non-recombining segments and the nearly full-length genome of HBoV2 using the best-fitting models. The results indicated that the evolutionary rates of non-recombinant fragments ranged from 6.4 × 10^−5^–3.3 × 10^−4^ substitutions/site/year, and the tMRCAs ranged from 1,558 to 1,873 (Table S4). Correspondingly, the tMRCAs of NS1, NP1, and VP1 were traced back to 1866, 1947, 1730, and 1759, respectively (Table S5). Notably, the evolutionary rate and tMRCA of the nearly full-length genome of HBoV2 fell within this range.

### Selective pressure analysis of HBoV2

The selective pressure analysis of the three coding regions, NS1, NP1, and VP1 of HBoV2, revealed that all ω-values were less than 1, indicating that these regions were under negative selective pressure, with their evolutionary process primarily shaped by purifying selection. Among the three genes, the NP1 gene exhibited the highest ω-value (0.30) within the range of 0.15–0.30, suggesting it experienced greater selective pressure compared to the other two genes (Table 2).

**TABLE 2:** The number of selective pressure sites in the three coding regions NS1, NP1, and VP1 of HBoV2 included in this study^*a*^

Gene	Mean dN/dS (ω)	SLAC		FUBAR		FEL		MEME	Total^*b*^	
		PSS	NSS	PSS	NSS	PSS	NSS	PSS	PSS	NSS
NS1	0.15	0	14	2	50	0	40	6	1	37
NP1	0.30	0	3	0	11	0	13	0	0	11
VP1	0.20	0	38	1	99	0	109	7	0	89

^*a*^ PSS, positively selection site; NSS, negatively selection site.

^*b*^ Selective pressure site inferred by at least two of four algorithms.

A site-by-site analysis of the three coding regions, NS1, NP1, and VP1, of HBoV2 included in this study identified one PSS at codon 635 in the NS1 gene, detected by at least two of the four algorithms (SLAC, FUBAR, FEL, and MEME). However, no PSS was found in the NP1 and VP1 genes. Additionally, multiple NSSs were identified by at least two algorithms: 37 NSSs in the NS1 gene, 11 NSSs in the NP1 gene, and 89 NSSs in the VP1 gene (Table 2 and Table S6).

## DISCUSSION

In this study, the prevalence of HBoVs in children with acute gastroenteritis in Beijing, China, in about 10 years varied considerably from 0.6% to 3.8% with an overall of 2.5% and peaks in 2014 and 2015 and troughs in 2020 and 2022. This indicated that the HBoV infection varied from year to year (from 0.6% to 3.8%) in the range of 2.2%–20.4% in China (13, 14, 26), which may be attributed to the virus’s transmission dynamics, environmental factors, or alterations in host immune status. Moreover, a seasonal pattern of HBoVs infection was observed in Beijing, particularly between 2018 and 2021, with increased positive rates from August to December, aligning with previous research conducted in China (14, 27). Most HBoVs-positive patients were under 5 years of age, accounting for 98.7% (78/79), especially in children aged 6 to 24 months (65.8%, 52/79), which might be correlated with the deficiency of the immune system in this age group.

Among four genotypes of HBoVs (HBoV1-4), HBoV1 has been reported as the dominant one in respiratory infections, while HBoV2-4 were associated with acute gastroenteritis. In this study, HBoV4 was not detected. Among HBoV1-3, HBoV2 (65.8%) was the dominant genotype among pediatric patients with acute gastroenteritis in Beijing, supported by reports from other areas of China (12, 13, 26).

Phylogenetic analysis of the 15 nearly full-length HBoV2 sequences revealed that HBoV2C (86.7%, 13/15) was the predominant genotype of HBoV2 in Beijing, China, and exhibited greater conservation compared to HBoV2A. There were two sequences (FB1682-HBoV2-China-2017 and FB1686-HBoV2-China-2017) clustered into HBoV2A. The recombination analyses revealed that the two HBoV2A sequences were undergoing new intra-genotype recombination events, with recombination breakpoints in the NS1 and VP3 gene regions, respectively. The recombination event of FB1686-HBoV2-China-2017 with breakpoints in the VP3 gene was similar to that of GU301644-HBoV2-2007 and KM624025-HBoV2-2012 from China and GU048662-HBoV2-2007 from Thailand. In recent years, several epidemiological studies monitoring the genetic diversity of HBoVs have identified potential recombination events, with intra-genotype recombination playing a significant role in the evolution of HBoV2 (10, 28). Therefore, recombination may be the main source for high genetic variability and faster rate of evolution of HBoV2.

The evolutionary rate of HBoV1 has been widely reported, but there was no report on the evolutionary rate of the nearly full-length sequences of HBoV2 (29, 30). The BEAST analysis estimated a global tMRCA for HBoV2 dating back to 1845, with a mean nucleotide substitution rate of 1.4 × 10⁻⁴ substitutions/site/year for nearly full-length sequences, which is faster than that of HBoV1, highlighting the need for ongoing, enhanced monitoring of HBoV2 (29, 31). In addition, HBoV2A diverged earlier and evolved at a faster rate than HBoV2C and had higher nucleotide diversity, which suggested that HBoV2A was undergoing rapid evolution. Moreover, the NP1 gene of HBoV2 showed a faster evolution rate at the nucleotide level instead of the amino acid level, which was consistent with previous reports (29, 30).

Selection pressure analysis revealed that the ω-values for the three HBoV2 genes were all less than 1, indicating that they were under purifying selection pressure. A previous study reported a strong correlation between selective pressure and evolutionary rate in B19 viruses (32). In this study, selection pressure and evolutionary rate were proportional for the three genes of HBoV2, and the NP1 gene was under the strongest selection pressure. Site-by-site analysis identified a PSS at codon 635 in the NS1 gene, located within the middle helicase domain, but outside the four conserved Walker motifs that are responsible for 3'−5' helicase activity. However, its specific role requires further investigation (6).

There were several limitations that should be acknowledged. Firstly, this is single-center research with a limited number of clinical specimens. Secondly, all specimens were collected from outpatients who lacked detailed clinical data, such as the progression and the outcome of disease, which limited the building of the correlation between HBoVs infection and the severity of acute gastroenteritis. Thirdly, due to the low number of specimens positive for HBoV2 in children with acute gastroenteritis, only 15 nearly full-length genomes of HBoV2 were harvested in this study, which may introduce bias in phylogenetic analyses. Finally, the lack of a large number of nearly full-length sequences of HBoV2 in GenBank and the high-frequency intra-genotype recombination of HBoV2 limited the estimation of evolutionary rate and tMRCA in this study. These limitations could be improved in the future with the accumulated complete genome sequences.

In conclusion, HBoV2 was the dominant genotype in children with acute gastroenteritis in Beijing, China, and children younger than 2 years are more susceptible to HBoVs. Novel recombination events between HBoV2 sub-genotypes occurred frequently, which explained the high genetic variability and a faster rate of evolution of HBoV2. More data should be accumulated to confirm these results.

## Data Availability

The nearly full-length genome sequences of HBoV2 obtained in this study were deposited in the GenBank database under accession numbers PQ728256–PQ728270.

## SUPPLEMENTAL MATERIAL

Supplemental material spectrum.03327-24-s0001.pdf Tables S1 to S6; Fig. S1 to S3.

ASM does not own the copyrights to Supplemental Material that may be linked to, or accessed through, an article. The authors have granted ASM a non-exclusive, world-wide license to publish the Supplemental Material files. Please contact the corresponding author directly for reuse.
