# Supplementary material for: Prevalence and molecular characterization of human bocavirus in children with acute gastroenteritis in Beijing, China, during 2014–2023
Source: Microbiol Spectr. 2025 Apr 25;13(6):e03327-24. doi: 10.1128/spectrum.03327-24 (PMC12131841; doi:10.1128/spectrum.03327-24)
Supplement: Supplemental material — Tables S1 to S6; Fig. S1 to S3. [file spectrum.03327-24-s0001.pdf]

Supplementary Materials

Table S1. Primers designed to amplify the nearly full-length genome sequences of HBoV2.

Target gene	Primers	Sequence (5’ -3’)	Product (bp)
NS1	NS1F1	TGCCGGCAGACATATT	
	NS1R1	AGGAGAGATCAACCGATT	918
	NS1F2	ATACAGAGACAAGCGAGGTG	
	NS1R2	TAAACACTCCTCCCACCA	844
	NS1F3	GCTTTTATGGTCCTGCTT	
	NS1R3	TCTCTTCTTGGATGGACG	797
NP1	NP1F	ATCGTCCATCCAAGAAGA	
	NP1R	AAAGCATTTCTTCGTCTG	723
VP1	VP1F	TTGGGATGATGTCTACCG	
	VP1R	ACCCACACCAGAACCTTT	602
VP2	VP2F1	ATAACGAGCCTAAACCAG	
	VP2R1	AATGTATGCTCTTTCGTT	788
	VP2-S-F	GAAGACGCAAATGCTGTA	
	VP2-S-R	CTGTGTTTCCGTGCTGTC	226
	VP2F2	GACAGCACGGAAACACAG	
	VP2R2	ATGCCTGACGCAGTACAA	1035

Table S2. Reference sequences downloaded from GenBank for the construction of phylogenetic trees.

Accession no.	Genotype	Country	Year
FJ496754	HBoV1	China	2003
DQ000495	HBoV1	Sweden	2005
DQ000496	HBoV1	Sweden	2005
DQ340570	HBoV1	USA	2005
DQ457413	HBoV1	China	2006
DQ778300	HBoV1	China	2006
DQ988933	HBoV1	China	2006
DQ988934	HBoV1	China	2006
GQ926982	HBoV1	China	2008
FJ375127	HBoV1	China	2008
FJ695472	HBoV1	Germany	2008
AB480175	HBoV1	Japan	2009
KX373884	HBoV1	Mexico	2010
JN128955	HBoV1	China	2011
JN387081	HBoV1	China	2011
JX887482	HBoV1	China	2012
MH828524	HBoV1	Vietnam	2013
KU557406	HBoV1	Egypt	2014
MF376170	HBoV1	Belarus	2014
MH828530	HBoV1	Vietnam	2014
KY629421	HBoV1	Argentina	2015
MG953829	HBoV1	Brazil	2015
MF374982	HBoV1	USA	2016
MG383449	HBoV1	Ethiopia	2016
MN887275	HBoV1	China	2017
OL519573	HBoV1	China	2018

Accession no.	Genotype	Country	Year
LC720423	HBoV1	Japan	2020
LC671301	HBoV1	Japan	2021
MZ468527	HBoV1	Croatia	2021
LC769220	HBoV1	Japan	2022
EU082213	HBoV2	Australian	2001
EU082214	HBoV2	Australian	2001
FJ948860	HBoV2	Australian	2001
FJ973558	HBoV2	Tunisia	2006
FJ973559	HBoV2	Nigeria	2007
FJ973560	HBoV2	Nigeria	2007
GU048662	HBoV2	Thailand	2007
GU048663	HBoV2	Thailand	2007
GU301644	HBoV2	China	2007
GU301645	HBoV2	China	2007
FJ170278	HBoV2	Pakistan	2008
FJ170279	HBoV2	Pakistan	2008
FJ375129	HBoV2	China	2008
GU048664	HBoV2	UK	2008
FJ170280	HBoV2	UK	2009
GQ200737	HBoV2	Pakistan	2009
JQ964116	HBoV2	Russia	2010
JX257046	HBoV2	China	2011
KM624025	HBoV2	China	2012
KY050744	HBoV2	Vietnam	2013
MG953831	HBoV2	Brazil	2014
MF680549	HBoV2	South Korea	2016
MG383447	HBoV2	Ethiopia	2016
MG383450	HBoV2	Ethiopia	2016
MZ546192	HBoV2	China	2016
EU918736	HBoV3	Australia	2001
FJ973563	HBoV3	Nigeria	2007
FJ973562	HBoV3	Nigeria	2007
OP255991	HBoV3	Netherlands	2018
FJ973561	HBoV4	Nigeria	2007
KC461233	HBoV4	Thailand	2011
MG383446	HBoV4	Ethiopia	2016

Table S3. Bayes factor comparison of six models for HBoV2.

Date	Best-fitting model	ln BF	Model 1	Model 2	Model 3	Model 4	Model 5	Model 6
HBoV2 complete genome	HKY +F+I+G4	Model 1	-	1.9	23.1	-136.	-35.9	-40.2
		Model 2	-1.9	-	21.3	-138.9	-37.8	-42.06
		Model 3	-23.1	-21.3	-	-159.1	-59.1	-63.3
		Model 4	136.0	137.9	159.1	-	100.1	95.8
		Model 5	35.9	37.8	59.1	-100.1	-	-4.3
		Model 6	40.2	42.1	63.3	-95.8	4.3	-

Date	Best-fitting model	ln BF	Model 1	Model 2	Model 3	Model 4	Model 5	Model 6
HBoV2A complete genome	HKY +F+I	Model 1	-	0.1	-1.2	-88.3	-85.0	-88.0
		Model 2	-0.1	-	-1.4	-88.1	-85.2	-88.1
		Model 3	1.2	1.4	-	-87.1	-83.8	-86.7
		Model 4	88.3	88.4	87.1	-	3.2	0.32
		Model 5	85.0	85.2	83.8	-3.2	-	-2.9
		Model 6	88.0	88.1	86.7	-0.3	2.9	-
HBoV2C complete genome	TN +F+I	Model 1	-	1.5	1.8	-16.0	-17.8	-17.4
		Model 2	-1.5	-	0.3	-17.4	-19.2	-18.8
		Model 3	-1.8	-0.3	-	-17.7	-19.6	-19.2
		Model 4	16.0	17.4	17.7	-	-1.9	-1.4
		Model 5	17.8	19.3	19.6	1.9	-	0.4
		Model 6	17.4	18.8	19.2	1.4	-0.4	-
HBoV2 NS1	TN +F+I	Model 1	-	2..0	4.4	0.3	5.5	1.4
		Model 2	-2.0	-	2.4	-1.7	3.4	-0.6
		Model 3	-4.4	-24	-	-4.1	1.1	-3.0
		Model 4	-0.3	1.7	4.1	-	4.2	1.1
		Model 5	-5.5	-3.4	-1.1	-5.2	-	-4.1
		Model 6	-1.4	0.6	3.0	-1.1	4.1	-
HBoV2 NP1	HKY +F+I	Model 1	-	2.0	4.8	-8.3	-3.4	-9.7
		Model 2	-2.0	-	2.8	-10.4	-5.4	-11.7
		Model 3	-4.8	-2.8	-	-13.2	-8.2	-14.5
		Model 4	8.3	10.4	13.2	-	4.9	-1.3
		Model 5	3.4	5.4	8.2	-4.9	-	-6.2
		Model 6	9.7	11.7	14.5	1.3	6.2	-
HBoV2 VP1	HKY +F+I+G4	Model 1	-	2.3	2.7	-64.9	-59.9	-55.6
		Model 2	-2.3	-	0.4	-67.2	-62.2	-57.9
		Model 3	-2.7	-0.4	-	-67.6	-62.6	-58.3
		Model 4	64.9	67.2	67.6	-	5.0	9.3
		Model 5	59.9	62.2	62.6	-4.9	-	4.29
		Model 6	55.6	57.9	58.3	-9.3	-4.3	-

Date	Best-fitting model	ln BF	Model 1	Model 2	Model 3	Model 4	Model 5	Model 6
HBoV2 VP3	HKY +F+I+G4	Model 1	-	1.2	1.0	-63.7	-60.6	-63.8
		Model 2	-1.2	-	-0.2	-64.9	-61.8	-65.1
		Model 3	-1.0	0.2	-	-64.8	-61.6	-64.9
		Model 4	63.7	64.9	64.8	-	3.2	-0.1
		Model 5	60.6	61.8	61.6	-3.2	-	-3.3
		Model 6	63.8	65.1	64.9	0.1	3.3	-

BF: Bayes Factor, ln BF = ln *P* (D|Model1)-ln *P*(D|Model2), If BF > 3, the data provide support for model 1 over model 2.

Model 1: Strick clock + Constant size, Model 2: Strick clock + Exponential growth, Model 3: Strick clock + Bayesian skyline plot, Model 4: Uncorrelated relaxed clock + Constant Size, Model 5: Uncorrelated relaxed clock + Exponential growth, Model 6: Uncorrelated relaxed clock + Bayesian skyline plot.

Table S4. Evolutionary analysis of non-recombining fragments of HBoV2.

Recombination event	NO. of sequences	Region	Mean Evolutionary Rate	95% HPD	tMRCA
FB1682-HBoV2-China-2017	30	1-4218 nt	6.4×10^{-5}	1.5×10^{-7} -1.5×10^{-4}	1558
		4219-5100 nt	2.1×10^{-4}	2.6×10^{-5} -4.8×10^{-4}	1763
FB1686-HBoV2-China-2017	18	1-1935+ 4001-4487 nt	3.3×10^{-4}	1.4×10^{-6} -7.6×10^{-4}	1865
		1936-4000+ 4488-5100 nt	2.2×10^{-4}	1.6×10^{-7} -5.5×10^{-4}	1873
HBoV2C	31	1-3164 nt	8.4×10^{-5}	1.7×10^{-6} -1.8×10^{-4}	1787
		3165-5100 nt	2.0×10^{-4}	1.4×10^{-5} -4.0×10^{-4}	1789

Table S5. Evolutionary analysis of four proteins of HBoV2 after accounting for the recombination breakpoints

Protein	Mean Evolutionary Rate	95% HPD	tMRCA
NS1	1.3×10^{-4}	5.2×10^{-5} - 1.9×10^{-4}	1866
NP1	2.4×10^{-4}	2.5×10^{-6} - 4.8×10^{-4}	1947

Protein	Mean Evolutionary Rate	95% HPD	tMRCA
VP1	1.5×10^{-4}	3.9×10^{-5} - 2.6×10^{-4}	1730
VP3	1.8×10^{-4}	3.1×10^{-5} - 3.6×10^{-4}	1759

Table S6. Selection pressure sites inferred by at least two of four algorithms in the three coding regions NS1, NP1, and VP1 of HBoV2 included in this study.

Gene	PSS	NSS
NS1	635	11,16,21,31,104,108,127,166,193,217,265,295,355,362,368,370, 371,390,399,400,401,406,408,415,416,418,423,428,434,464,468, 476,486,495,549,589,629
NP1	N/A	16,81,132,133,137,139,151,154,164,169,181
VP1	N/A	26,47,50,59,64,122,127,132,134,138,139,141,143,146,150,152, 153,154,156,171,178,179,187,193,195,204,217,218,223,227,235, 239,248,249,251,252,260,262,263,269,293,300,303,305,306,317, 329,339,341,345,347,356,358,363,374,401,403,409,417,431,449, 455,461,480,492,498,504,507,509,519,526,545,547,549,551,553, 572,573,574,598,620, 625,630,634,640,647,650,653,663

PSS: Positively selection site; NSS: Negatively selection site.

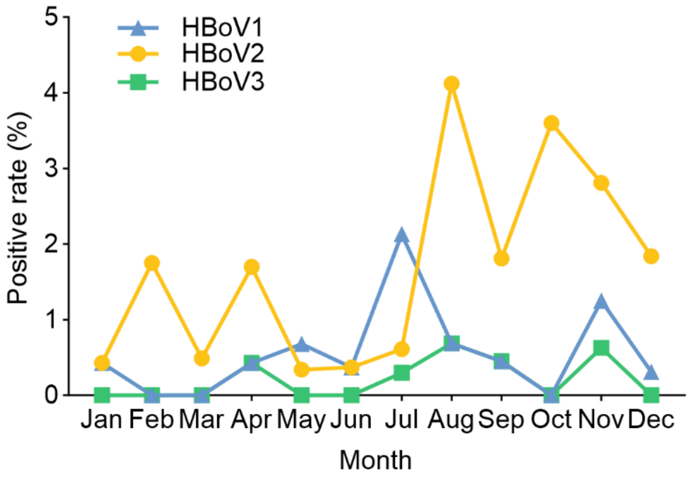

Fig S1. Changing monthly distribution of HBoVs genotypes detected in children with acute gastroenteritis in Beijing, China from 2014 to 2023. Each line indicates the distribution of each genotype.

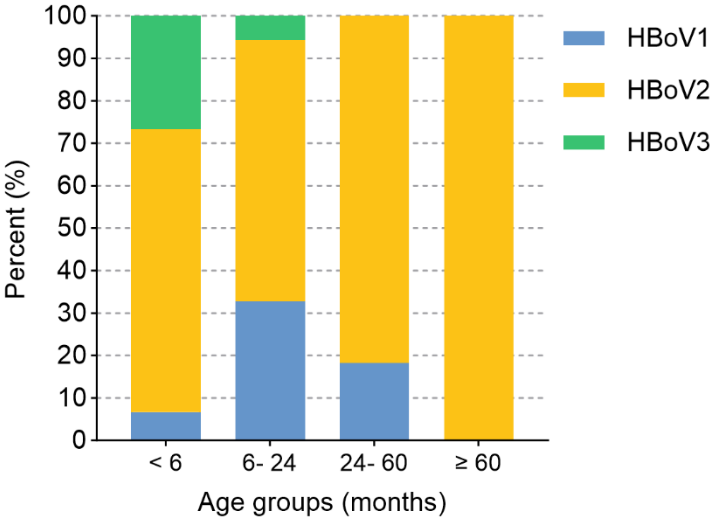

Fig S2. Distribution of HBoVs genotypes in various age groups of children with acute gastroenteritis in Beijing, China from 2014 to 2023.

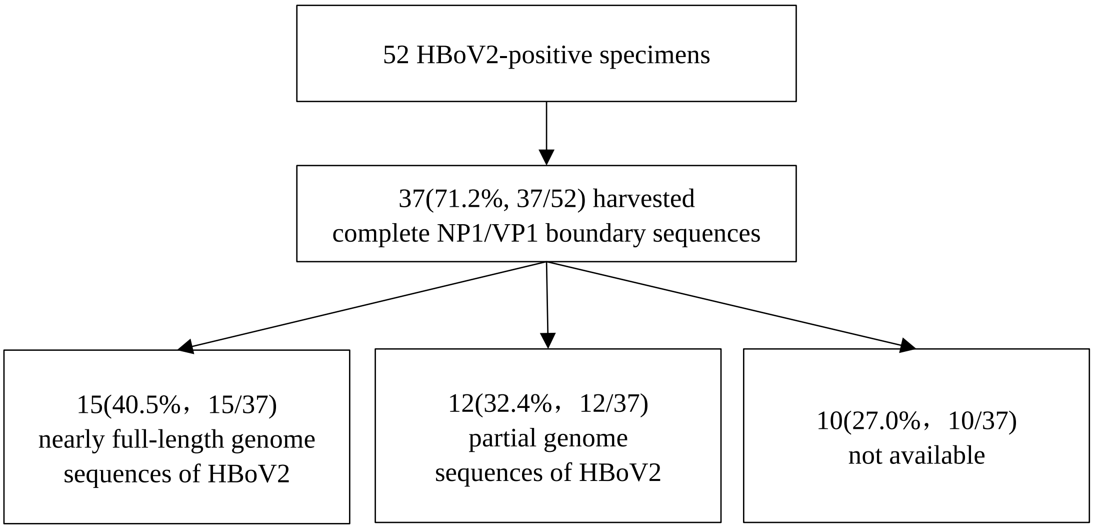

Fig S3. The flow chart of this study
